# Effect of rehabilitation on renal outcomes after acute kidney injury associated with cardiovascular disease: a retrospective analysis

**DOI:** 10.1186/s12882-024-03666-z

**Published:** 2024-07-12

**Authors:** Yuma Hirano, Tomoyuki Fujikura, Kenichi Kono, Tomoya Yamaguchi, Naro Ohashi, Yurina Yokoyama, Masahiro Toda, Katsuya Yamauchi, Hideo Yasuda

**Affiliations:** 1 Department of Rehabilitation Medicine, Hamamatsu University Hospital, Hamamatsu City Shizuoka, Japan; 2 Internal Medicine 1, Hamamatsu University School of Medicine, Hamamatsu City Shizuoka, Japan; 3 Department of Physical Therapy, International University of Health and Welfare School of Health Science at Narita, Narita City Chiba, Japan

**Keywords:** Acute kidney injury, Cardiovascular disease, Rehabilitation, Renal outcome

## Abstract

**Background:**

Acute kidney injury (AKI) incidence is extremely high worldwide, and patients who develop AKI are at increased risk of developing chronic kidney disease (CKD), CKD progression, and end-stage kidney disease (ESKD). However, there is no established treatment strategy for AKI. Based on the idea that exercise has a stabilizing effect on hemodynamics, we hypothesized that rehabilitation would have beneficial renal outcomes in patients with AKI associated with cardiovascular disease. Therefore, the purpose of this study was to determine whether rehabilitation can stabilize hemodynamics and positively impact renal outcomes in patients with AKI associated with cardiovascular disease.

**Methods:**

In total, 107 patients with AKI associated with cardiovascular disease were enrolled in this single-center retrospective study and were either assigned to the exposure group (*n* = 36), which received rehabilitation at least once a week for at least 8 consecutive weeks, or to the control group (*n* = 71). Estimated glomerular filtration rate was assessed at baseline before admission, at the lowest value during hospitalization, and at 3, 12, and 24 months after enrolment. Trends over time (group × time) between the two groups were compared using generalized estimating equations. Moreover, congestive status was assessed by amino-terminal pro-B-type natriuretic peptide (NT-proBNP), and the effect of rehabilitation on congestion improvement was investigated using logistical regression analysis.

**Results:**

The time course of renal function after AKI, from baseline to each of the three timepoints suggested significant differences between the two groups (*p* < 0.01). However, there was no significant difference between the two groups at any time point in terms of percentage of patients who experienced a 40% estimated glomerular filtration rate reduction from that at baseline. The proportion of patients with improved congestion was significantly higher in the exposure group compared with that in the control group (*p* = 0.018). Logistic regression analysis showed that rehabilitation was significantly associated with improved congestion (*p* = 0.021, OR: 0.260, 95%CI: 0.083–0.815).

**Conclusion:**

Our results suggest that rehabilitation in patients with AKI associated with cardiovascular disease correlates with an improvement in congestion and may have a positive effect on the course of renal function.

## Background

In addition to its high incidence, acute kidney injury (AKI) is associated with cardiovascular events [1], reduced quality of life [2, 3], mortality [4], and subsequent decline in renal function [5, 6] with an increased risk of progression to chronic kidney disease (CKD), CKD progression, and end-stage kidney disease (ESKD) [7, 8]. This is also true for mild stage 1 AKI [9, 10], and there is a need to establish therapeutic guidelines to mitigate the decline in renal function in patients after they develop AKI.

In particular, AKI associated with cardiovascular disease involves a specific set of symptoms encompassing cardio-renal syndrome, which has attracted attention in recent years and is frequently encountered in daily medical practice. In fact, AKI occurs in 27–40% of the patients hospitalized for acute decompensated heart failure [11], and AKI associated with cardiovascular disease is associated with worse renal outcomes [12]. The possibility of a pathophysiological mechanism resulting from hemodynamic changes rather than true renal injury is relevant, given the close association of cardiac and renal failure due to hemodynamic abnormalities. Renal outcome after AKI is influenced not only by decreased left ventricular function and cardiac output but also by congestion as a hemodynamic factor [13, 14]. Congestion increases renal venous pressure, which increases renal interstitial pressure and causes vascular injury to the peri-interstitial peritubular capillaries. This results in pressure drainage of the tubular lumen, leading to an increase in tubular luminal pressure, and a decrease in glomerular filtration rate (GFR) due to a lower glomerular filtration pressure.

Exercise may improve the hemodynamics, averting worse renal outcomes in patients who develop AKI associated with cardiovascular disease. Three months of exercise in patients with acute myocardial infarction has been reported to reduce systemic vascular resistance via improvements in nitric oxide levels [15]. Exercise further improves sympathovagal balance and contributes to hemodynamic stabilization in patients with heart failure [16].

In summary, worse renal outcomes after AKI are associated with hemodynamic changes, which can be stabilized by exercise. However, no studies have investigated the effects of rehabilitation on renal outcomes after AKI in humans. We hypothesized that rehabilitation may improve congestion through hemodynamic stabilization and have a positive impact on renal outcomes in patients who develop AKI. In this study, we conducted a retrospective analysis of patients with cardiovascular disease who developed AKI during hospitalization to evaluate the efficacy of rehabilitation.

## Methods

### Selection and allocation of patients

This retrospective observational study included adult patients aged ≥ 18 years with cardiovascular disease who were admitted to the Department of Cardiology, Cardiovascular Surgery, and Nephrology at Hamamatsu University Hospital between January 2016 and July 2020 and who developed AKI during their hospitalization. AKI was diagnosed based on the Kidney Disease Improving Global Outcomes criteria [17]. The study included patients who had AKI at the time of hospital admission and those who developed AKI during the period of hospitalization. Patients with missing baseline renal function assessments, unassessed renal function, hospital transfers, or those who died during the follow-up period were excluded from the analysis. The patients included in the final analysis were divided into the following two groups: control and exposure groups. The exposure group comprised patients who underwent rehabilitation during hospitalization after the onset of AKI and continued supervised rehabilitation at least once a week after discharge for at least 8 weeks. Patients who underwent rehabilitation for less than 8 weeks or less than once a week under supervision were included in the control group. There are a few published reports on the renoprotective effects of rehabilitation on human AKI. Based on previous studies that reported the renoprotective effects of aerobic exercise in rats and previous studies that verified the rehabilitation effects on human CKD, we set the supervised rehabilitation period at 8 weeks [18–20]. Patient characteristics for each group were extracted from the medical record data (Table 1). This study adhered to the "Strengthening the Reporting of Observational Studies in Epidemiology (STROBE)" Guidelines.

**Table 1:** Baseline characteristics of study participants

	Control group (*n* = 71)	Exposure group (*n* = 36)	*p-*Value
Age (year)	70 (65–76)	73 (66–80)	0.282
Sex [N, (%)]			
Male	44 (62.0)	23 (63.9)	0.846
Female	27 (38.0)	13 (36.1)	
BMI (kg/m^2^)	23.8 (20.5–26.4)	25.1 (22.5–28.0)	0.098
Current smoking [N, (%)]	14 (20.6)	5 (13.9)	0.400
Median length of hospital stay (days)	20 (14–29)	30 (19–51)	0.001^*^
Walking independence at discharge [N, (%)]	65 (91.5)	36 (100)	0.095
Main reason for admission [N, (%)]			
Worsening HF	25 (35.2)	11 (30.6)	0.630
Ischemic heart disease			
CABG	2 (2.8)	2 (5.6)	0.413
PCI	4 (5.6)	10 (27.8)	0.002^*^
Valvular disease			
TAVI	0 (0)	1 (2.8)	0.336
Surgical treatment	13 (18.3)	2 (5.6)	0.073
Aortic disease			
Conservative treatment	4 (5.6)	0 (0)	0.188
Surgical treatment	7 (9.9)	4 (11.1)	0.542
Arrhythmia	6 (8.5)	2 (5.6)	0.456
Cardiomyopathy	3 (4.2)	1 (2.8)	0.587
Others	7 (9.9)	3 (8.3)	0.550
Medication [N, (%)]			
Loop diuretic	55 (77.5)	26 (72.2)	0.550
ACE inhibitor	8 (11.3)	8 (22.2)	0.133
ARB	38 (53.5)	17 (47.2)	0.538
Beta blocker	63 (88.7)	30 (83.3)	0.310
MRA	22 (40.0)	12 (33.3)	0.805
SGLT2 inhibitor	5 (7.0)	3 (8.3)	0.544
LVEF < 40% [N, (%)]	16 (22.5)	18 (50.0)	0.004
NT-proBNP (pg/mL)	2247 (605–5422)	2909 (1339–6175)	0.314
Hypertension [N, (%)]	64 (90.1)	33 (91.7)	0.550
Hyperlipemia [N, (%)]	44 (62.0)	29 (80.6)	0.051
Diabetes mellitus [N, (%)]	42 (59.2)	25 (69.4)	0.299
Stroke [N, (%)]	35 (49.3)	17 (47.2)	0.839
Unscheduled mechanical ventilation [N, (%)]	12 (16.9)	16 (44.4)	0.002^*^
Sepsis [N, (%)]	4 (5.6)	8 (22.2)	0.010^*^
CKD stage [N, (%)]			
G1	0 (0.0)	1 (2.8)	
G2	24 (33.8)	10 (27.8)	
G3a	23 (32.4)	11 (30.6)	0.537
G3b	14 (19.7)	11 (30.6)	
G4	10 (14.1)	3 (8.3)	
AKI stage [N, (%)]			
Stage 1	58 (81.7)	28 (77.8)	
Stage 2	8 (11.3)	6 (16.7)	0.479
Stage 3	5 (7.0)	2 (5.6)	

Data are expressed as percentages in parentheses or as medians (IQR). *p*-values were calculated using the unpaired *t*-test, Mann–Whitney U test, χ^2^ test, or Fisher’s exact test

Patients with FIM-walking scores of 6 or 7 were defined as walking independently

NT-proBNP data are shown for 87 patients measured during hospitalization and 3 months after the onset of AKI

AKI stage mentioned in Table 1 represents the maximum stage during hospitalization

*BMI* body mass index, *HF* heart failure, *CABG* coronary artery bypass grafting, *PCI* percutaneous coronary intervention, *TAVI* transcatheter aortic valve implantation, *ACE* angiotensin converting enzyme, *ARB* angiotensin II receptor blocker, *MRA* mineralocorticoid receptor antagonist, *SGLT2* sodium glucose cotransporter 2, *LVEF* left ventricular ejection fraction, *NT-proBNP* amino-terminal pro-B-type natriuretic peptide, *CKD* chronic kidney disease, *AKI* acute kidney injury

### Exposure

Standard rehabilitation at our hospital followed the guidelines set forth by the Japanese Society of Cardiology and the Japanese Society of Cardiac Rehabilitation. During hospitalization, early mobilization was initiated according to the overall condition of the patient, followed by aerobic exercise and resistance training for 5–6 days per week. Upon discharge, patients could participate in outpatient cardiac rehabilitation. Those who agreed to participate were transferred to an outpatient rehabilitation program.

**Fig. 1:**
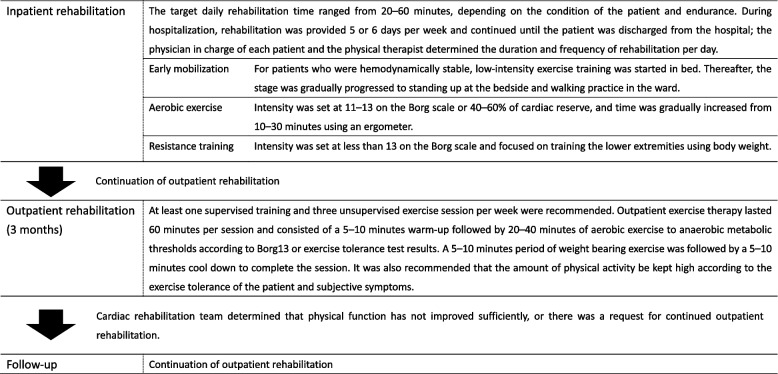
Description of the rehabilitation for the exposure group. Depending on the general condition of each patient, standard rehabilitation was performed according to the guidelines of the Japanese Society of Cardiology and the Japanese Society of Cardiac Rehabilitation

In outpatient settings, patients were advised to maintain a high level of physical activity based on their exercise tolerance and subjective symptoms. The recommended outpatient rehabilitation duration was three months. At the end of this period, motor function was evaluated to assess the extent of functional improvement. If a satisfactory improvement was observed, the rehabilitation program was concluded. However, if the cardiac rehabilitation team determined that there was insufficient improvement or if the patient requested continued outpatient rehabilitation, the duration of the program was extended accordingly (The details of the rehabilitation are shown in Fig. 1).

### Endpoint

The primary endpoint of this study was the estimated GFR (eGFR) over a 2-year period following AKI onset. The eGFR data were collected at baseline; at the lowest value during hospitalization; and at 3, 12, and 24 months after AKI. The eGFR was calculated using the Japanese estimation formula based on serum creatinine concentration [21]. Baseline renal function was determined as the latest stable serum creatinine and eGFR up to 12 months before the onset of the disease, in accordance with the findings of the previous studies [7]. As a secondary endpoint, the percentage of patients who experienced a 40% eGFR reduction from that at baseline was assessed. This is considered as a surrogate endpoint associated with the risk of ESKD in Japanese patients with CKD [22]. This risk was assessed at 12 and 24 months after the onset of AKI. Further, amino-terminal pro-B-type natriuretic peptide (NT-proBNP) was monitored during hospitalization and at 3 months after the onset of AKI as an indicator of improvement in congestion. NT-proBNP measured during the hospitalization period was the highest. Improvement in congestion was defined as a ≥ 30% decrease in NT-proBNP from hospitalization to 3 months after AKI onset [23].

### Ethical considerations

To ensure patient privacy and confidentiality, each participant was assigned a study ID number, which was used for data collection and analysis. All collected data were securely stored in a designated location with appropriate measures to protect their integrity and confidentiality. This study was approved by the Clinical Research Ethics Committee of Hamamatsu University School of Medicine (Study No. 22–200). Patient consent was not required as this study was based on publicly available data.

### Statistical analysis

Statistical analyses were performed using IBM SPSS Statistics version 26.0. Continuous variables are presented as either mean and standard deviation or median and interquartile range (IQR), and comparisons between groups were performed using either unpaired t-tests or Mann–Whitney U tests, depending on the data distribution. Categorical variables are expressed as frequencies and percentages and were compared using the chi-square (χ^2^) test. Generalized estimating equations (GEE) with a Gaussian distribution and identity link function were employed to assess the effect of rehabilitation on changes in renal function at each time point. The GEE models were adjusted for multiplicity to account for repeated measures and included the following variables: group (control group or exposure group), time (at Baseline, Lowest level, at 3 months, at 12 months, and at 24 months), and the interaction term (Group × Time). Further, we adjusted for age, sex, loop diuretic, renin-angiotensin system inhibitor, length of hospital stay, and AKI stage. The selection of adjustment variables was based on data from previous studies [24, 25] and medical expertise of the clinicians. Logistic regression analysis was used to compare the percentage of patients who experienced a 40% eGFR reduction from that at baseline at 12 and 24 months. A 40% eGFR reduction from that at baseline served as the dependent variable, whereas exposure was the independent variable. Logistic regression models were adjusted for the same variables as those used in the GEE models. In addition, ancillary analysis was performed on patients with measurable NT-proBNP during hospitalization and at 3 months. A chi-square (χ^2^) test was performed to compare the proportion of patients with improved congestion from hospitalization to 3 months after AKI between the control and exposure groups. In addition, logistical regression analysis was performed with improvement in congestion as the dependent variable and rehabilitation status as the independent variable, adjusted for age and use of loop diuretics. The threshold for statistical significance was set at *p* < 0.05.

## Results

### Analysis of participants and differences in patient characteristics between the two groups

**Fig. 2:**
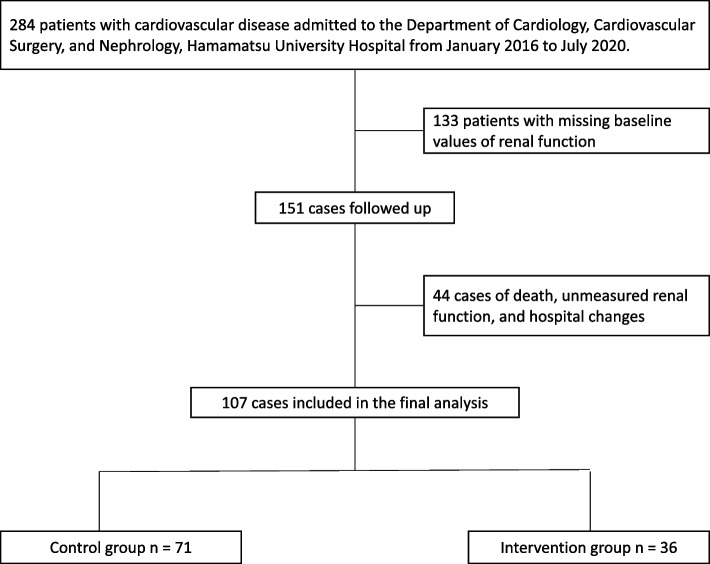
Selection of participants for analysis and assignment to the two groups

A total of 2918 patients were hospitalized for cardiovascular disease during the study period; of these, 284 (9.7%) developed AKI during hospitalization. Of the 284 patients with cardiovascular disease who developed AKI during hospitalization, 107 were included in the final analysis after excluding 177 patients with missing baseline and post-AKI follow-up records. Among the included patients, 36 (33.6%) were assigned to the exposure group and received continuous rehabilitation at least once a week for a minimum of 8 weeks, while 71 (66.4%) were assigned to the control group (Fig. 2). The patients in the exposure group received continuous rehabilitation for a median of 16 weeks (IQR:12–24), whereas those in the control group received it for a median of two weeks (IQR:1–3). There were no exercise-related adverse events such as new cardiac arrhythmias, hemodynamic instability, or self extubation in either group. The patients in the exposure group had a significantly longer median length of hospital stay than those in the control group (*p* < 0.01). Additionally, the patients in the exposure group underwent percutaneous coronary intervention (*p* < 0.05), unscheduled mechanical ventilation during hospitalization (*p* < 0.05), and sepsis (*p* < 0.05) at a significantly higher rate than those in the control group. Among the patients included in the analysis, 106 (99.1%) had concurrent CKD. One patient required temporary dialysis during the hospitalization period. In addition, one patient transitioned to maintenance dialysis during the 24-month observation period. More than 90% of patients in both groups were ambulatory and independent at the time of discharge (Table 1).

### Comparison of renal function trends after the onset of AKI between the two groups

**Fig. 3:**
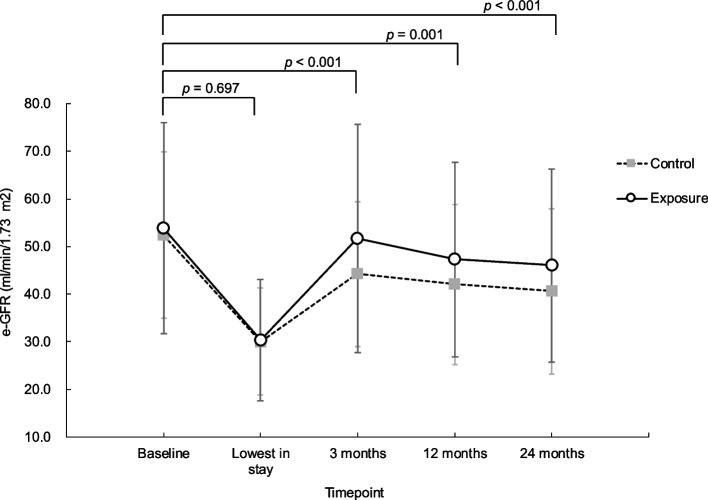
Trends in renal function after AKI in each group. AKI, acute kidney injury; e-GFR, estimated glomerular filtration rate

Renal function decreased over time in the entire patient population from baseline to all time points (*p* < 0.001). There was no significant interaction effect between the group and time on the transition of renal function from baseline to the lowest value during hospitalization. However, there was a significant interaction effect between group and time from baseline to the 3-, 12-, and 24-month timepoints (Fig. 3 and Table 2).

**Table 2:** Changes in renal function over time

	Marginal means												
	Baseline		Lowest			3 months			12 months			24 months	
Control group	52.5 (17.5)		30.0 (11.2)			44.2 (15.3)			42.1 (16.8)			40.6 (17.4)	
Exposure group	53.9 (22.2)		30.3 (12.8)			51.8 (24.0)			47.3 (20.5)			46.0 (20.2)	
	Group Fixed effect	Time						Group × Time					
		Baseline Vs Lowest		Baseline Vs 3 months	Baseline Vs 12 months		Baseline Vs 24 months	Baseline → Lowest		Baseline → 3 months	Baseline → 12 months		Baseline → 24 months
*p*-value	0.939	< 0.001		< 0.001	< 0.001		< 0.001	0.697		< 0.001	0.001		< 0.001

The following data are exhibited for each group at Baseline; Lowest during the stay; and at 3, 12, and 24 months: estimated glomerular filtration rate trends, the main effect of group, the main effect of time, and the interaction effect between the group and time. We used a generalized estimating equation to examine changes in the measures over time. Each model was adjusted for age, sex, loop diuretic, renin-angiotensin system inhibitor, length of hospital stay and AKI stage. Values of renal function are expressed as means and standard deviations

### Forty percent eGFR reduction from baseline after AKI

**Fig. 4:**
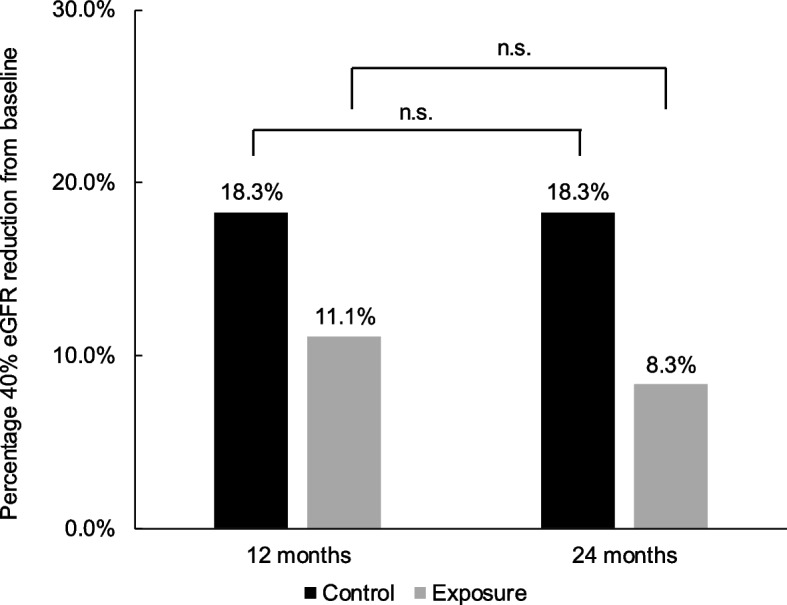
Comparison of the percentage of patients at 40% eGFR reduction from baseline. There was no difference in the percentage of patients at 40% eGFR reduction from baseline at each time point

In the control group, at both 12 and 24 months, 18.3% of the patients experienced a 40% eGFR reduction from that at baseline. In comparison, 11.1% of the patients in the exposure group experienced a 40% eGFR reduction from that at baseline to the 12-month timepoint and 8.3% from baseline to the 24-month timepoint (Fig. 4). However, logistic regression analysis indicated that the rehabilitation did not have a significant effect on mitigating 40% eGFR reduction from that at baseline at either 12 or 24 months (12 months: p = 0.253, odds ratio [OR]:2.384, 95% confidence interval [CI]:0.537–10.573; 24 months: p = 0.127, OR:3.321, 95% CI:0.712–15.484).

### Improvement in congestion from hospitalization to 3 months after AKI

Ancillary analysis included 87 patients (control group; *n* = 55, exposure group; *n* = 32) with measurable NT-proBNP during hospitalization and at 3 months after AKI onset. The proportion of patients with improved congestion was significantly higher in the exposure group [84.4% (*n* = 27)] compared with in the control group [60.0% (*n* = 33)] (p = 0.018). Logistic regression analysis adjusted for age and use of loop diuretics showed that rehabilitation was significantly associated with improved congestion (p = 0.021, OR: 0.260, 95%CI: 0.083–0.815).

## Discussion

In this study, we tested the efficacy of rehabilitation on renal outcomes after AKI associated with cardiovascular disease. To further support this validation, the impact of rehabilitation on improving congestion associated with renal outcomes was also examined.

There was a significant interaction between groups and time at 3, 12, and 24 months after AKI onset. This suggests that rehabilitation after AKI may have a positive effect on renal function trends. In addition, the rehabilitation status was associated with improvement in congestion at 3 months. We believe that congestion is a factor that may mediate rehabilitation and renal outcomes, thus increasing the validity of the results of this study. However, there were no significant differences in the clinically important endpoint, i.e., the percentage of patients who experienced a 40% eGFR reduction from that at baseline between the two groups at any time point, suggesting that the effect may have been limited. Animal studies that reported that exercise reduces the decline in renal function after AKI [26] support the biological validity of our results. However, few reports have examined the effect of exercise after AKI on renal function decline in humans, and our results represent a novel finding with a potential for further clinical implications.

In this study, rehabilitation appeared to be significantly associated with recovery of renal function 3 months after the onset of AKI. It is hypothesized that this positive impact may have persisted up to the timepoint of 12 and 24 months thereafter. There are different patterns of recovery of renal function after AKI, some cases show gradual recovery over weeks to months after AKI onset, others show recovery within a few days after AKI onset [27, 28]. One of the results of this study, the renal function trend was seen to have a positive impact in the exposure group at the 3-month timepoint from baseline, and NT-proBNP at 3-month time point was significantly improved in the exposure group. Since exercise improves NT-proBNP in patients with cardiovascular disease [15, 16, 29–31], and since congestion is implicated in renal outcomes after AKI [13, 14], rehabilitation may contribute to the renal tissue repair process at 3 months after AKI by improving congestion through hemodynamic stabilization. In fact, in this study, a clear difference was observed in the mean eGFR (mL/min/1.73 m^2^) values from baseline to 3 months, with values of -8.3 in the control group and -2.1 in the exposure group. However, from 3 to 12 months, the difference was -2.1 for the control group and -4.5 for the exposure group. While the difference tended to decrease gradually from 12 to 24 months, with values of -1.5 for the control group and -1.3 for the exposure group. A previous study reported that if patients did not progress to CKD 3 months after the onset of AKI, the cumulative incidence of CKD in subsequent patients was comparable to that of patients who did not develop AKI [32]. In other words, the results suggest that it may be clinically important to promote the recovery of renal function by providing rehabilitation for 3 months after AKI onset.

While approximately 80% of the study participants had mild stage 1 AKI, most patients had concurrent CKD. It has been reported that pre-existing CKD has a negative effect on renal function recovery after AKI [33, 34]. It has also been reported that the development of hypertension and proteinuria is accelerated after AKI in CKD model rats [35], and it is well known that hypertension can be improved by exercise. In other words, the participants in this study represent a population in which the effect of rehabilitation on renal outcomes was easily observed; however, it is unclear whether the results of this study can be extrapolated to all patients with AKI.

A previous meta-analysis indicated that patients with AKI had an 8.8-fold higher risk of renal function decline than those without AKI [5]. However, the percentage of patients who experienced a 40% eGFR reduction from that at baseline in this study was 11.1% at 1 year and 8.3% at 2 years in the exposure group despite the development of AKI. The outcome is similar to that reported in a Japanese CKD case cohort study (4.3% at 1 year and 10.9% at 2 years) [22]. This suggests that rehabilitation after AKI may be an add-on therapy to improve renal outcomes and prevent CKD progression; however, further long-term effectiveness needs to be verified.

Despite extensive research into the development of therapeutic and preventive agents for AKI, treatment strategies for AKI in human patients remain undetermined. One of the primary reasons for this that AKI affects a diverse patient population, showing variations in background factors, comorbidities, and AKI severity, challenging the establishment of effective treatment approaches. In this study, the causes of AKI varied among different cardiovascular diseases. Therefore, rehabilitation has invaluable benefits for this diverse patient population. Considering the diverse nature of patients who develop AKI in clinical practice, these findings hold great promise for future clinical applications.

This study had some limitations. First, since this study was a single-center study, the sample size was small and a large number of patients had incomplete baseline data. Hence, the study findings may have a low generalizability and external validity. Second, as this was a retrospective study, there may have been a selection bias with regard to the allocation between the two groups. The patients in the exposure group, on account of adhering to the rehabilitation regimen, may have been inherently more health consciousness and determined to maintain a high level of health status. This, in turn, may have contributed to the reduction in renal function decline. Previous studies have indicated that high self-care and disease knowledge scores are associated with a reduced risk of renal function decline in patients [36]. Therefore, it remains uncertain whether these results may be applied to patients who have a low awareness of exercise and physical activity. In addition, the retrospective nature of the study makes it difficult to determine whether patients dropped out of rehabilitation for personal reasons, and thus the reasons for noncompliance with rehabilitation is unknown. Third, the exercise regimen for the exposure group was not rigidly structured. Although the guidelines were followed, the study’s retrospective nature led to variations in the content of the rehabilitation for each participant. Therefore, we defined rehabilitation as an intervention performed at least once a week for a minimum of 8 weeks to unify the frequency and period of intervention. Finally, the eGFR may have been influenced by muscle mass. A comparison of patient characteristics revealed that the patients in the exposure group had longer hospital stays and higher rates of unscheduled mechanical ventilation and sepsis than those in the control group, which was indicative of a higher proportion of critically ill patients. Since eGFR calculated from serum creatinine is sensitive to muscle mass, it is possible that the exposure group with more critically ill patients had an overestimated eGFR due to decreased muscle mass. However, most patients in the exposure group (35/36; 97.2%) maintained walking independence throughout the 2-year observation period. Walking independence is associated with trunk muscle mass, psoas major, and sarcopenia [37–39]. Therefore, we considered that no appreciable loss of muscle mass was observed over the 2-year period, and the effect on eGFR and the bias related to muscle mass was minimal.

## Conclusion

Our findings suggest that rehabilitation for patients with AKI may help reduce the decline in their renal function after AKI onset. However, the rehabilitation was insufficient to prevent a 40% eGFR reduction from that at baseline. In particular, it may be essential to rehabilitate for 3 months following AKI to help restore renal function. It is important to conduct future prospective studies that focus on more specific and detailed intervention methods, as well as the optimal duration of intervention, to effectively mitigate long-term renal function decline. By addressing these aspects, we can further enhance our understanding and improve the effectiveness of rehabilitation strategies for patients with AKI.

## Data Availability

The data presented in this study are available on request from the corresponding author. The data are not publicly available because they are the property of the Institute of Hamamatsu University Hospital, Japan.

## Abbreviations

AKI: Acute kidney injury
BNP: Brain natriuretic peptide
CKD: Chronic kidney disease
ESKD: End-stage kidney disease
GEE: Generalized estimating equations
GFR: Glomerular filtration rate
NT-proBNP: Amino-terminal pro-B-type natriuretic peptide
